# Implementing a Sodium-Glucose Cotransporter 2 Inhibitor Module With a Software Tool (Future Health Today): Qualitative Study

**DOI:** 10.2196/50737

**Published:** 2024-03-13

**Authors:** Matthew Suen, Jo-Anne Manski-Nankervis, Caroline McBride, Natalie Lumsden, Barbara Hunter

**Affiliations:** 1 Department of General Practice and Primary Care University of Melbourne Parkville Australia

**Keywords:** type 2 diabetes, CP-FIT, electronic health, clinical decision support tool, primary care, SGLT2 inhibitor, complication, tool, digital health intervention, thematic analysis, decision support, diabetes management

## Abstract

**Background:**

Primary care plays a key role in the management of type 2 diabetes. Sodium-glucose cotransporter 2 (SGLT2) inhibitors have been demonstrated to reduce hospitalization and cardiac and renal complications. Tools that optimize management, including appropriate prescribing, are a priority for treating chronic diseases. Future Health Today (FHT) is software that facilitates clinical decision support and quality improvement. FHT applies algorithms to data stored in electronic medical records in general practice to identify patients who are at risk of a chronic disease or who have a chronic disease that may benefit from intensification of management. The platform continues to evolve because of rigorous evaluation, continuous improvement, and expansion of the conditions hosted on the platform. FHT currently displays recommendations for the identification and management of chronic kidney disease, cardiovascular disease, type 2 diabetes, and cancer risk. A new module will be introduced to FHT focusing on SGLT2 inhibitors in patients with type 2 diabetes who have chronic kidney diseases, cardiovascular diseases, or risk factors for cardiovascular disease.

**Objective:**

The study aims to explore the barriers and enablers to the implementation of an SGLT2 inhibitor module within the Future Health Today software.

**Methods:**

Clinic staff were recruited to participate in interviews on their experience in their use of a tool to improve prescribing behavior for SGLT2 inhibitors. Thematic analysis was guided by Clinical Performance Feedback Intervention Theory.

**Results:**

In total, 16 interviews were completed. Identified enablers of use included workflow alignment, clinical appropriateness, and active delivery of the module. Key barriers to use were competing priorities, staff engagement, and knowledge of the clinical topic.

**Conclusions:**

There is a recognized benefit to the use of a clinical decision support tool to support type 2 diabetes management, but barriers were identified that impeded the usability and actionability of the module. Successful and effective implementation of this tool could support the optimization of patient management of type 2 diabetes in primary care.

## Introduction

Type 2 diabetes places a significant burden on both people with this condition and the Australian health system. An estimated 1.3 million Australians older than the age of 15 years have diabetes [1], with an associated Aus $14 billion (US $12.9 billion at 2010 rates) [2] of health spending. This creates an enormous social and economic burden. General practitioners (GPs) play a vital role in reducing the impact of diabetes as most people receive their medical care in general practice. Guidelines produced by the Australian Diabetes Society [3], The Royal Australian College of General Practitioners [4], and the Living Evidence for Diabetes Consortium [5] provide support to GPs to inform both pharmacological and nonpharmacological management decisions.

Sodium-glucose cotransporter 2 (SGLT2) inhibitors have been included in Australian guidelines for years, but their place in therapy has evolved as recent evidence demonstrates secondary prevention benefits for cardiovascular disease (CVD) and chronic kidney disease (CKD) irrespective of glycated hemoglobin [6-11]. Prescribing of SGLT2 inhibitors remains low [12], and reported barriers include a lack of knowledge of its nonglycemic benefits and concerns about side effects [13]. This can be mitigated if appropriate and relevant education is available [14]. However, education alone is not likely to have a significant impact on changing prescribing behavior.

Future Health Today (FHT) is a co-designed quality improvement and clinical decision support technology platform aimed at the detection and management of conditions in general practice. FHT integrates with the electronic medical record to provide two components: (1) a clinical decision support tool active at the point of care (PoC) and (2) a web-based dashboard that facilitates practice-wide audit and quality improvement activities, including patient recall. Wraparound activities are also included with links to the latest guidelines and access to educational resources. Following initial optimization in 12 general practices, using guidelines for CKD, CVD, and cancer prevention, it is now in use in 55 general practices across Australia. A new module was introduced to FHT practices in July 2022 recommending SGLT2 inhibitor prescribing in patients with type 2 diabetes who also have CKD, CVD, or risk factors for CVD consistent with the Australian Evidence-Based Clinical Guidelines for Diabetes [5]. If a patient is suitable for prescribing, the statement “Consider initiation of SGLT2 inhibitor to reduce CVD and CKD risk” will appear in the PoC. The objective of this paper is to report on the evaluation of the implementation of this new SGLT2 inhibitor module in FHT.

## Methods

### Study Design

Fifty-two practices in Victoria (n=51) and Tasmania (n=1) had access to the SGLT2 module between July 2022 and January 2023. A qualitative evaluation exploring the use of the module was undertaken using the Clinical Performance Feedback Intervention Theory (CP-FIT), a theory for designing, implementing, and evaluating feedback [15]. CP-FIT was chosen as it incorporates and builds on 30 pre-existing theories and was developed specifically for the health care context. The theory proposes 42 variables that influence a feedback cycle with each step vital for successful feedback to occur. The theory postulates that the cycle is affected by 3 variables: feedback, recipient, and context. In the context of FHT, CP-FIT outlines the steps that users move through when guideline-based recommendations are communicated to users. Algorithms are applied to electronic medical records (data collection and analysis); recommendations are delivered to users (feedback); and they are received (interaction), interpreted (perception), and interrogated (verification). If users find the recommendations appropriate (acceptance), they will respond to them (intention and behavior) and ultimately leads to changes in patient care (clinical performance improvement).

Participants were recruited through expression of interest to participate in semistructured interviews, from those who had at least 1 month access to the SGLT2 inhibitor module. Expressions of interest were sent via email to practices encouraging participation in an interview. Advertisements in the department and practice-based research network newsletters were also used to promote the study. Interviews were conducted between September and December 2022. All participants had access to the FHT software for between 6 and 18 months prior to the use of the SGLT2 inhibitor module in clinical practice.

### Data Collection

Semistructured interviews using CP-FIT as a guide were conducted with GPs, general practice nurses (GPNs), and clinical health assistants (CHAs) to explore their perspectives on using the module and recommendations for improving the tool. CHA are administrative staff who use patient data for health research and quality improvement. The aim was to gather as many perspectives as possible until saturation was reached. Participants participated in one-to-one interviews that occurred via telephone or Zoom (Zoom Technologies). Interviews were conducted by MS, a male academic GP registrar with the University of Melbourne. An interview guide was developed to focus on questions regarding the clinical usefulness of the recommendations, the impact on clinical workflow, and perceived changes in clinical performance. Data were uploaded to NVivo (version 12; QSR International) and coded by MS to identify themes using CP-FIT as a coding framework. A second researcher (CM) reviewed the data to ensure the reliability of the analysis.

### Ethical Considerations

This study was approved (23269) by the Faculty of Medicine, Dentistry and Health Sciences Human Ethics Subcommittee at The University of Melbourne. Consent was taken through a signed consent form prior to the interview, and verbal consent was also gained to record the audio component of the interview. Participation was voluntary, and participants were compensated with an Aus $50 (US $32) voucher for their time. Participants’ names and practice locations were deidentified and replaced by a pseudonym only known to MS and CM to ensure the protection of their privacy.

## Results

### Overview

Invitations for interviews were sent out to clinics that had at least 1 month of experience with the SGLT2 inhibitor module. There was interest from 14 general practice clinics, and 16 interviews were completed with participants from 11 clinics (Table 1). The sex of the interviewee was confirmed during the interview. A further 3 interviews were not completed as the interviewees used FHT and previous modules but not the SGLT2 inhibitor module. Interviews ranged from 15 to 42 minutes.

**Figure 1:**
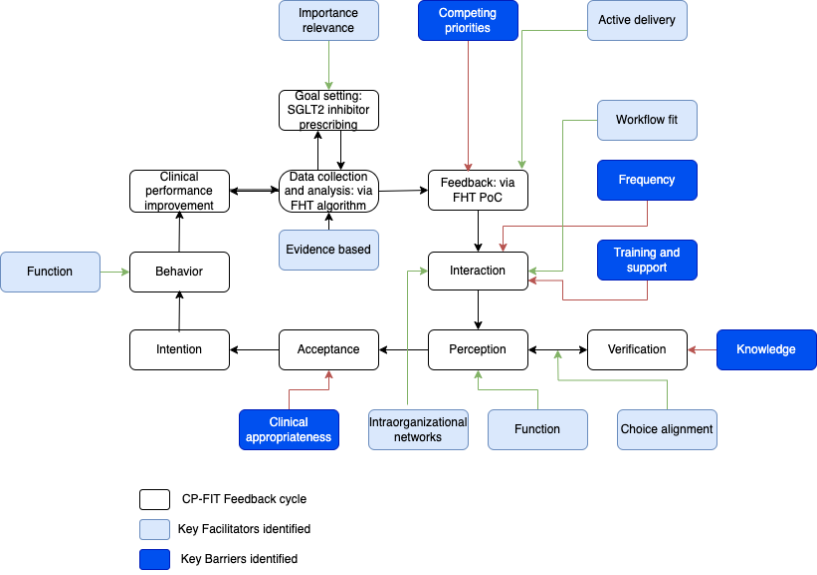
How CP-FIT explains the effectiveness of the FHT intervention. CP-FIT: Clinical Performance Feedback Intervention Theory; FHT: Future Health Today; PoC: point of care; SGLT2: sodium-glucose cotransporter 2.

The themes were mapped across the 3 variables: context, feedback, and recipient. Barriers and facilitators related to these variables are summarized in Figure 1.

**Table 1:** Characteristics of study participants (N=16).

			Participants, n (%)
**Role**			
	General practitioner	8 (50)	
	Practice nurse	6 (38)	
	Practice manager and practice nurse	1 (6)	
	Clinical health assistant	1 (6)	
**Sex**			
	Female	11 (69)	
	Male	5 (31)	
**Rurality of practice**			
	Metro	8 (50)	
	Regional or rural or remote	8 (50)	

### Feedback Variables

#### Goal: Evidence-Based Nature, Relevance, and Importance

Common across all interviews were goals relating to the importance, relevance, and evidence-based nature of the SGLT2 recommendations. Users had confidence that the recommendations were accurate knowing they were based on the latest guidelines. Some incorporated FHT and the module in their conversation with patients by highlighting and explaining to patients the background and concept of FHT to instill patient confidence and acceptance of SGLT2 inhibitor prescribing.

I can say to people that the reason I’m suggesting the change in medicine or the increase in dose is because there is a little point of care prompt, it’s telling me there are advantages in doing this, in making a change.

GP2

#### Feedback Delivery: Active Delivery, Frequency, and Function

Active delivery was demonstrated through the PoC but not the dashboard. Participants found the PoC user friendly as it would automatically pop up once the patient file was opened. They also enjoyed the flexibility of being able to minimize and scroll through the recommendations in the PoC. In contrast, a majority indicated the need to log in to the dashboard to access it was a barrier to its use.

Well it [PoC] comes up automatically every time we enter a patient where it's relevant and it's a very good prompt to double check that we're following the guidelines.

GP8

Practices using the SGLT2 inhibitor module also had access to over 20 recommendations within FHT relating to other chronic diseases. With the multiple modules available, prompt fatigue was highlighted as a barrier, especially with time constraints during consultations. Several participants highlighted the possibility of a behavioral norm of not checking the PoC recommendations when they pop up. Despite this, most still believed the recommendations were helpful in identifying patients who would not have been highlighted if it was not for the prompt, reiterating the tool as an additional safety net.

It comes up on every patient when we open the file but because we’re so busy during a consultation...and it comes up so often.

GP2

### Context Variables

#### Organization and Team Characteristics: Workflow Fit, Intraorganizational Networks, and Competing Priorities

Many users reported using the PoC function before or during the consultation to guide clinical decision-making when talking broadly about FHT. They indicated that they referred to it when the consultation was diabetes focused but would otherwise flag it for future reference or disregard it. “I have a look at the point of care and see what kind of recommendations are popping up” (GP1).

The SGLT2 module encouraged collaboration between the staff. With the use of the cohort function, CHA and GPNs were able to generate recall list and book appointments for GPs to review patient suitability. This reduced the workload for GPs. In addition, GPNs felt comfortable initiating a conversation about the benefits of the medications, although they also acknowledged the final decision lay with the GP.

The autonomy is given to be able to do recalls without having to have the GP absolutely check through every little detail of the patient.

CHA1

Competing priorities were identified as barriers to fully use FHT with a flow on effect to the SGLT2 inhibitor module, especially with the use of the dashboard. Multiple GPs commented that they used the PoC as the recommendations came to them once the patient file was open. This is in contrast with the dashboard which involved creating patient lists, recalling patients, and filling quality improvement forms. Time pressure was highlighted as a barrier, with many GPs commenting about having time constraints and an increased in workload due to workforce shortages, particularly in rural and regional clinics.

As with all of those things in a busy practice, the catch is putting time aside [to use FHT]. I never get around to doing so.

GP8

Participants believed a lack of familiarity and knowledge with SGLT2 inhibitors hindered their use of the module. This stemmed from a lack of time for users to upskill. They were aware of the education resources and support offered through FHT, but time pressures did not allow them to review these resources. For the feedback cycle to be completed, GPs must show leadership in engaging with the module as they are the ones that make the decision on whether the recommendation is clinically appropriate. Practice staff have commented that due to time constraints and competing priorities, GPs were unable to prioritize the use of FHT.

I think our GPs, you need to actually show them and go through it with them [FHT], then there’s always that “I’m time poor, not right now, can’t do it too much.”

CHA1

#### Patient Population: Clinical Appropriateness and Choice Alignment

Participants recognized that the recommendations were a guide only and still required the use of clinical acumen. Although they acknowledged the accuracy, they may not be clinically appropriate at the individual patient level due to factors such as age, health literacy, comorbidities, and likely compliance.

They’re [GPs] not willing to introduce stricter control for a 70 year old that’s got diabetes and he’s well controlled and everything else.... It’s just a little bit reassessment and the individualisation of the knowing your patients.

Practice nurse 7

#### Implementation Process: Cost, Training, and Support

The main cost attributed to the use of FHT was time. For GPs, the time spent on the use of PoC before and during consultations was appropriate for the benefit given as the recommendations could be actionable at the time or delayed. However, the dashboard was unused by GPs as they did not have time to use it. In contrast, practice nurses and the CHA found the dashboard function useful to create recall lists and as a quality improvement tool.

Would I love to have more of an opportunity to look at the online module and setting up cohorts and doing things? I’d love to have more time doing that.

GP2

Participants reported that more support and training would increase their familiarity of confidence in discussing and prescribing SGLT2 inhibitors. They were aware of the educational materials offered to them through FHT, but time constraints prevented them from using them. Practice nurses noted that the resources provided specifically for the SGLT2 inhibitor module were tailored more to GPs for prescribing with broader education on the topic likely to be more beneficial for them. While feedback for these resources was positive, participants believed other modes of education would be beneficial for the broader use of FHT.

I suppose maybe just some how to put it into practice, maybe. Like some scenarios or that type of thing. See while these patients come in, this has popped up, this is what we should be talking about and discussing.

Practice nurse 6

### Recipient Variables

Many found it useful to prompt and raise awareness of the benefits of prescribing SGLT2 inhibitors but found the overcapture of patients an issue. While commenting they were confident with the accuracy of the recommendations, the guideline-based recommendations did not take the patient’s personal history and suitability into account. Participants believed that to maximize the potential of the module, they are still needed to use their own clinical judgment to interrogate and decide if the recommendations were appropriate. Some commented that by following the recommendations blindly, it would reduce the “patient centredness” of the clinical decision and remove shared decision-making with the patient. “I wouldn't have thought of it without the popup.... It’s making medicine more recipe-like” (GP3).

After seeing the recommendations, users identified their own need for further education on SGLT2 inhibitors and diabetic management in general. This stemmed from the need for more knowledge on the topics for them to confidently discuss these medications with patients. While they were aware of the resources offered through FHT, time constraints for education were again noted.

It’s improved my ability...my little areas of where I need to hone my education skills… I probably didn’t do it that well until we had the popup that forced me to think about why am I telling this patient.

Practice nurse 2

The recommendation is shown every time the patient file is opened independent of the reason for presentation. Users have commented that it would be difficult to introduce the recommendations if the patients presented for a nondiabetes-related consultation and to steer the conversation toward diabetes. Some users would take note of it and often suggest making a subsequent appointment to discuss SGLT2 inhibitor prescribing. Others used the recommendation as a prompt to change their consultation style to incorporate the module in their consults.

If I was to see a diabetic who wasn’t on a SGLT2 and had cardiovascular risks, then I would - I would in time change my plan. But as you would well know, best practice is hard to change because you’ve got to learn a new mental routine.

GP6

## Discussion

### Principal Findings

The overall response to the use of the SGLT2 inhibitor module in FHT was positive. Enablers and barriers to the use of this module were explored. Enablers of the module’s use included users’ confidence about the accuracy of the recommendations and that they found it easy to incorporate into appropriate consultations and comfortable introducing the topic to patients. They felt the recommendations were proactive and a useful prompt for users to consider prescribing the medication. Unfamiliarity with SGLT2 inhibitors is a known barrier to its prescription [16], and GPs’ awareness of the nonglycemic benefits is low [14]. The inclusion and presentation of SGLT2 inhibitors in guidelines are relatively new, and they have also encouraged users to upskill on knowledge for them to feel more comfortable discussing it with patients.

The role of the GP and GPN and both vital to the management of diabetes in the primary care setting [17], and the module has helped define these roles. GPNs used the module to create lists from the dashboard to recall patients to initiate a conversation about possible SGLT2 inhibitor prescribing and optimization of diabetes management in general with the final decision made by the GP to determine suitability. The main barrier to the use of the module was time pressure and competing priorities during the consultations. Many users stated that the heavy workload prevented them from using the full function of FHT.

By mapping against the CP-FIT framework, the PoC function allowed users to move through the feedback cycle successfully. It has led to better recordkeeping (data collection and analysis) to maximize the efficacy of the algorithms to produce accurate recommendations. They still required to use their clinical acumen to decide whether the recommendation was appropriate for the patient (feedback, interaction, and perception). If appropriate (acceptance), they would initiate a conversation with the patient about prescribing SGLT2 inhibitors and discussing the benefits of improved glycemic control and better patient outcomes (clinical performance improvement). The expanding number of modules has led to prompt fatigue causing users to reduce their interaction with PoC and not initiating the feedback cycle.

There were several limitations to this study. Approximately half of the users of this module were the practice champions for FHT in their workplace. This may lead to responder bias with their enthusiasm for FHT making them more likely to participate and have a better understanding of the program. The study was conducted during the COVID-19 pandemic, which may prevent potential users from engaging with the program as the effects of the pandemic caused disruption to workflow, especially with staff and resource shortages. We aim to conduct follow-up interviews in the future to see if the pandemic had any significant effect on the use of FHT.

The use of clinical decision support systems (CDSSs) in diabetes has previously been shown to improve patient outcomes [18], although studies are focused on the hospital setting and glycemic control [19,20]. Literature has consistently reported a gap between optimal diabetes care practice and recommended care standards. Previous CDSS studies have found high clinician satisfaction with its use to facilitate the intensification of glucose control [21]. There is strong evidence that users of CDSS consistently recommend its use to others [22] and is appropriate for general practice use [23].

Previous barriers with CDSSs previously reported include increased workload and time constraints [24], which align with the findings of this study. A previous study showed that the implementation of a CDSS alone did not improve quality of care but required multifaceted strategies including continuing education and feedback mechanisms, organizational changes, and patient-orientated strategies [25]. While these strategies were made available to participants, engagement and uptake of the SGLT2 inhibitor module recommendations were variable.

With the increasing burden of type 2 diabetes on the Australian health care system, early diagnosis and treatment will no doubt reduce the risk of developing and delaying comorbidities and improve the quality of life for diabetics. CDSS can assist clinicians in diagnosing and optimizing the management of chronic disease beyond type 2 diabetes.

### Conclusions

This study highlights the benefit of a clinical decision support tool to improve appropriate prescribing and increase clinician awareness of SGLT2 inhibitors for their diabetic and cardiorenal effects. Successful implementation of this module could be used to detect patients who will benefit from the effects of SGLT2 inhibitors in primary care and assist in reducing all-cause mortality and morbidity with guideline-concordant prescribing.

## Abbreviations

CDSS: clinical decision support system
CHA: clinical health assistant
CKD: chronic kidney disease
CP-FIT: Clinical Performance Feedback Intervention Theory
FHT: Future Health Today
GP: general practitioner
GPN: general practice nurse
PoC: point of care
SGLT2: sodium-glucose cotransporter 2

## Data Availability

The data sets generated during this study are available from the corresponding author on reasonable request.
